# Experience with flash glucose monitoring: Impact on glycemic control and quality of life in type 1 diabetes within a public healthcare program

**DOI:** 10.1097/MD.0000000000045015

**Published:** 2025-10-10

**Authors:** Betyna Saldanha Corbal, Rafaela Flavia da Silva Puzic, Erica Tatiane da Silva, Thomaz Schröder Lameirinhas, Camille Moreira Baptista da Silva, Giovanna Saldanha Ostwald Corbal

**Affiliations:** a Fundação Oswaldo Cruz, Campus Universitário Darcy Ribeiro, Brasília – DF, Brazil; b Universidade de Brasília, Campus Universitário Darcy Ribeiro, Brasília – DF, Brazil; c Escola Superior de Ciências da Saúde, Setor Médico-Hospitalar Norte, Brasília – DF, Brazil.

**Keywords:** blood glucose, blood glucose meter, continuous glucose monitoring system, glycemic control, quality of life, self-monitoring of blood glucose, type 1 diabetes mellitus

## Abstract

Flash glucose monitoring (FGM) systems provide real-time interstitial glucose readings and offer a reliable alternative to capillary self-monitoring of blood glucose. This technology may improve diabetes management and quality of life (QoL) for individuals with type 1 diabetes mellitus. This prospective cohort study employed both qualitative and quantitative approaches to assess patient satisfaction, perceived user experience, glycemic control metrics, and QoL among adults with type 1 diabetes mellitus newly enrolled in the Federal District’s public FGM program in Brazil. Participants completed questionnaires, including the Brazilian version of the Diabetes Quality of Life Measure, at baseline and after 3 months of FGM use. Of the 83 enrolled patients, 69 completed the study, of whom 14 were excluded because of incomplete follow-up questionnaires. After 3 months of FGM use, the mean time in range was 68.96% and the mean glucose management indicator was 6.69%. A coefficient of variation of ≥ 36% was observed in 60.9% of the participants, with an average sensor scan frequency of 14.27 scans/day. The mean age was 33.8 years (SD = 9.4), and 50.7% of participants were female. Participants reported higher satisfaction with FGM than with self-monitoring of blood glucose, citing perceived improvements in comfort, practicality, diabetes management, and overall QoL. However, while QoL scores improved slightly after 3 months of FGM use, these changes were not statistically significant. FGM use was associated with high patient satisfaction and positive experiential feedback. Although QoL outcomes did not change significantly, the findings suggest perceived benefits in diabetes self-management among users.

## 1. Introduction

Brazil is the 3rd country with the most cases of type 1 diabetes mellitus (T1DM), behind the United States and India.^[1]^ Treating patients with diabetes mellitus aims to achieve optimal glucose management to minimize the risk of microvascular and macrovascular complications.^[2]^

Glycated hemoglobin (HbA1c) is the gold standard for assessing glycemic control and predicting the risk of chronic complications in T1DM, as demonstrated in a landmark clinical trial.^[3]^ Capillary self-monitoring of blood glucose (SMBG), traditionally recommended as 4 to 10 daily measurements, has been the primary strategy for lowering HbA1c levels.^[4]^ However, the invasiveness of this method makes it burdensome, leading to low adherence (approximately 44% of T1DM patients) due to needle fear, stigma, misconceptions, cost, and inconvenience.^[2,4]^

Technological innovations like flash glucose monitoring (FGM/FreeStyle Libre), have significantly reduced the daily challenges of managing T1DM, improving well-being and quality of life (QoL).^[2,5]^ Introduced in Europe (2014) and Brazil (2016), FGM uses a 14-day sensor for continuous glucose readings (similar to fingersticks) and has been shown to increase monitoring, reduce hypoglycemia, and improve QoL.^[6]^

While international studies have shown that FGM improves diabetes QoL,^[7]^ there is a paucity of research on this topic within the Brazilian context. The healthcare community increasingly agrees that patient-centered research is crucial for evaluating care, enhancing patient experience/QoL, and ensuring robust, relevant studies for more effective, patient-focused healthcare.^[8]^ Patient-centered research using patient-reported outcomes measured by replicable instruments known as patient-reported outcome measures^[8,9]^ is increasingly recognized as crucial for evaluating care and enhancing patient experience/QoL. For individuals with diabetes, the Diabetes Quality of Life (DQOL) instrument validated in Brazil as the DQOL-Brazil is one of the most established patient-reported outcome measures for assessing health-related QoL and changes following educational or pharmacological interventions, allowing both single-point and long-term assessments.^[10,11]^

In December 2020, the State Department of Health (SES/DF) implemented the pioneering Continuous Glucose Monitoring Program – Flash System for patients with T1DM within the Unified Health System (SUS),^[12]^ though its implementation remains limited to specific public services. However, the Brazilian Diabetes Society and other entities advocate nationwide FGM inclusion as standard care for both T1DM and type 2 diabetes mellitus.^[13]^ The National Commission for the Incorporation of Technologies in the Unified Health System held Public Call No. 42/2024 in June 2024 to evaluate the proposal to incorporate this technology through an analysis of the patient’s perspective.^[13]^

While most studies on continuous glucose monitoring (CGM) emphasize glycemic control and hypoglycemia prevention, this study aimed to evaluate patient-centered experiences and QoL related to FGM use in adults with T1DM within the SES/DF program.

## 2. Methods

This prospective mixed-methods cohort study evaluated the experience and QoL of T1DM patients in the SES/DF Program using FGM. Clinical and sociodemographic profiles, FGM metrics, user satisfaction, and QoL changes were analyzed to determine influencing factors.

Clinical profiles were characterized using the following variables: baseline HbA1c levels (<7% or ≥ 7%), diabetes duration (<5 years, 5–10 years, or > 10 years), insulin therapy regimen (multiple daily injections or continuous subcutaneous insulin infusion), insulin type, insulin dose per kilogram of body weight, bolus insulin percentage, presence of diabetes-related complications and/or comorbidities, and hypoglycemic events within 12 months before program enrollment. Sociodemographic characteristics included age (<20, 20–29, 30–39, 40–49, and ≥50 years), sex, race, residence region, and healthcare origin (public/private).

Clinical and sociodemographic data were obtained from the SES/DF digital database, which included procedural documents and completed questionnaires upon program entry.^[14]^

Prior FGM experience was also assessed. After 3 months in the SES/DF FGM program, the following LibreView data were used to analyze the device metrics: daily sensor scan frequency, percentage of sensor wear time, percentage of glucose readings < 54 mg/dL, percentage of glucose readings < 70 mg/dL, time in range (TIR; 70–180 mg/dL), percentage of glucose readings 181 to 250 mg/dL, percentage of glucose readings > 250 mg/dL, glucose management indicator, and coefficient of variation (CV%; <36% or ≥ 36%).

Patient FGM experience after 3 months was assessed via a Google Forms questionnaire, including: a 5-point Likert scale (1 = “very satisfied” to 5 = “very dissatisfied”) to evaluate satisfaction with FGM compared to SMBG; a question regarding the likelihood of recommending FGM to individuals with similar diabetes profiles (1 = “would always recommend” to 5 = “would never recommend”); and the extent to which they would be satisfied to continue using the technology, using a 5-point scale where 1 represents “very satisfied” and 5 represents “very dissatisfied. Additionally, open-ended questions were used to elicit participants” perceptions of the advantages and disadvantages of FGM.

In FGM users, QoL was measured at baseline and at the 3-month follow-up using DQOL-Brazil. This 44-item instrument comprises 4 domains: satisfaction (15 items), impact (18 items), social/vocational concerns (7 items), and diabetes-related concerns (4 items). Responses to each item were recorded on a 5-point Likert scale, where 1 corresponded to the most positive and 5 corresponded to the most negative QoL. Domain and total scores were calculated as the mean of individual item responses, where lower scores represent improved health-related QoL.^[11]^

Participants included adults (≥18 years) with T1DM newly enrolled in the SES/DF FGM Program who provided informed consent. The requirements for FGM use within the program were as follows: T1DM duration of at least 24 months; treatment with basal and rapid insulin analogs; documented SMBG at least 3 times daily in the preceding 6 months; attendance at diabetes education sessions; HbA1c ≤ 8% within the past 6 months; demonstrated proficiency in SMBG and carbohydrate counting; knowledge of hyperglycemia correction protocols (targets and insulin sensitivity factor); and knowledge of hypoglycemia correction protocols (values, symptoms, and treatment). Patients who did not complete the follow-up questionnaire within 3 to 6 months were excluded.

This study was conducted at specialized endocrinology outpatient clinics of the SES/DF, including the Specialized Center for Diabetes, Obesity, and Arterial Hypertension (CEDOH), Regional Hospital of Taguatinga (HRT), and Regional Hospital of Sobradinho between June 2022 and October 2023. Data were collected using questionnaires, administered in-person at HRT and CEDOH at the time of technology provision and digitally after 3 months. Written informed consent was obtained from all eligible participants.

Statistical analysis was performed by inputting data into a Microsoft Excel (Microsoft Corporation, Redmond) spreadsheet. Descriptive statistics were used for sociodemographic and clinical variables, closed-ended questions on experience, and QoL. Paired *t* tests or Wilcoxon tests were used to compare the QoL scores before and after FGM use. Ordinal logistic regression analysis was used to explore factors associated with QoL during FGM use, including sociodemographic and clinical profiles and FGM experience.

Statistical analyses were conducted using IBM SPSS Statistics for Windows version 22.0 (IBM Corp., Armonk), and normality was assessed using the Kolmogorov–Smirnov test with a significance level of 5%. Open-ended responses on the advantages and disadvantages of FGM were subjected to qualitative content analysis using Bardin method, involving pre-analysis, material exploration, and interpretation of results.

This study did not perform a priori sample size calculation. As a result, the number of participants may have limited the statistical power to detect significant differences in key outcomes, particularly changes in QoL. The absence of statistically significant results may, therefore, be partly attributable to a type II error.

## 3. Results

Of the 83 patients initially selected, 69 completed the study, of whom 14 were excluded because of incomplete follow-up questionnaires. The completed cohort included 2 pregnant women. The mean age of participants was 33.8 years (SD = 9.4), with a balanced gender distribution (50.7% female). The self-reported ethnicity was predominantly Brown (72.5%) and White (24.6%), with 2 Black participants. The majority resided in the central administrative region (36.2%), followed by the southwest (23.2%) and received care from public health services (56.5%). Detailed sociodemographic and clinical profiles are presented in Table 1.

**Table 1:** Sociodemographic and clinical profile of patients included in the study (N = 69).

Criteria			Frequency	Percentage
Age group (yr)	<20		2	2.9
	20–29		20	29.0
	30–39		23	33.3
	40–49		16	23.2
	≥50		8	11.6
Gender	Female		35	50.7
	Male		34	49.3
Self-reported ethnicity	White		17	24.6
	Black		2	2.9
	Mixed race		50	72.5
Health region of residence	Center-south		8	11.6
	Southwest		16	23.2
	Central		25	36.2
	West		2	2.9
	South		2	2.9
	North		15	21.7
	East		1	1.4
Type of service of origin	Public		39	56.5
	Private		30	43.5
HbA1c* at program enrollment	<7%		29	42.0
	≥7%		40	58.0
Diabetes duration	<5 yr		10	14.5
	5–10 yr		10	14.5
	>10 yr		49	71.0
Daily insulin units/ kg of weight	Mean (SDP)		0.74 (0.26)	–
Bolus percentage	Mean (DP)		54.75% (12.06)	–
Treatment	CSII		4	5.80
	MDI		65	94.20
	Basal insulin	Glargine	57	82.6
		Degludeca	7	10.1
		Ultra-rapid-acting insulin	3	4.3
		Regular	1	1.4
		Detemir	1	1.4
Diabetes-associated complications	No complications		58	84.1
	1 complication		5	7.2
	More than 1 complication		6	8.7
	Nephropathy		1	1.4
	Retinopathy		10	14.5
	Neuropathy		5	7.2
Other associated diseases	Hypothyroidism		12	17.4
	Celiac disease		1	1.4
	Dyslipidemia		4	5.8
	Hypertension		4	5.8
	Other		4	5.8
	No associated disease		44	63.8
Hypoglycemic emergency in previous 12 months	Yes		6	8.7
	No		63	91.3
** **Total			69	100

Source: author’s elaboration.

CSII = continuous subcutaneous insulin infusion, MDI = multiple daily injections, SD = standard deviation.

* HbA1c: glycated hemoglobin. Mean (SD) = 6.99% (0.81).

Of the participants, 62.32% reported prior FGM experience, with 26 originating from private healthcare services and 17 from public services. Table 2 presents the FGM metrics after 3 months of use. The analysis included 67 patients because 2 participants did not provide glucose metrics from LibreView.

**Table 2:** Technology metrics after 3 months of use (N = 67*).

Technology metric	Mean (SD)	Median (25th–75th percentile)	Minimum–maximum
Number of scans (views) per day	14.27 (9.57)	12.00 (7.00–18.00)	2–53
Sensor active time percentage	91.25 (13.98)	97.00 (89.00–100.00)	15–100
Percentage of glucose < 54 mg/dL	1.52 (2.04)	1.00 (00.00–3.00)	0–12
Percentage of glucose < 70 mg/dL	6.70 (4.49)	5.00 (3.00–9.00)	0–18
Time in range (TIR) (percentage of glucose 70–180 mg/dL)	68.96 (13.23)	70.00 (58.00–79.00)	34–93
Percentage of high glucose (181–250 mg/dL)	17.22 (9.72)	16.00 (10.00–23.00)	0–39
Percentage of very high glucose (>250 mg/dL)	5.55 (6.39)	4.00 (1.00–9.00)	0–40
Glucose management indicator (GMI)	6.69 (0.59)	6.60 (6.20–7.10)	5.6–8.7
Coefficient of variation of glucose†	37.69 (6.84)	37.60 (33.50–41.60)	25.2–56.5

Source: author’s elaboration.

GMI = glucose management indicator, SD = standard deviation, TIR = time in range.

* *Note*: 2 participants did not share their glucose metrics for LibreView.

† Forty-two participants (60.9%) had a coefficient of variation of glucose ≥ 36.

In terms of patient satisfaction, 65.2% reported being “very satisfied” with FGM compared to only 10.1% for self-monitoring of SMBG. Conversely, 7.2% were “very dissatisfied” with FGM and 29% with SMBG. Regarding continued FGM use, 88.4% were “very satisfied,” while 8.7% were “very dissatisfied.” Furthermore, 91.3% of patients stated that they “would always recommend” FGM to individuals with similar diabetes profiles, whereas 4.3% “would never recommend” it.

The qualitative findings are presented in Table 3, which categorizes the main themes derived from participants’ narratives concerning the perceived advantages (positive aspects) and disadvantages (negative aspects) of FGM use. The chart also reports the frequency of each theme, highlighting their relative prominence among participants.

**Table 3:** Advantages and disadvantages of SFGM use organized by themes.

Advantages		
Themes	Reports	Reported by (n)
Comfort	“It doesn’t hurt to apply the libre.” “Not having the pain of pricking your finger several times a day.”	5
Practicality and rapidity	“The practicality of measuring blood glucose anywhere. Inside public transport, walking down the street.” “The practicality of blood glucose medication, especially during physical activity.” “I can check my glucose all the time and quickly, with the libre my diabetes improved due to quick and practical measurement.”	4
Freedom	“The freedom to be able to check my blood glucose without interrupting the activities I am performing.” More freedom, more autonomy, more security and more comfort in treatment.” “Greater food freedom; greater quality and freedom of life, in general.”	4
Discretion in glucose measurement	“In prolonged work meetings it is also a peace not having to ask/ warn that I will have to leave to check blood glucose. I measure and no one even notices. I don’t disturb anything.” “Ease of measuring at any time discreetly.”	2
Improved glucose monitoring	“Having more control of how blood glucose is evolving during the day, according to what you eat, exercise. A more continuous control.” “It is much easier to make any correction in blood glucose, and constant use shows how the body reacts to each insulin or activity.” “There are no limits to checking blood glucose.” Greater predictability and ability to anticipate blood glucose control. Unlimited possibility of checking glucose.” “Being able to monitor my blood glucose and hypoglycemia much better, I have never fainted from hypo since I started using it, I had already fainted with hypo while driving and I live alone with my daughter who is a baby so with the use of the sensor I am much calmer especially at dawn.” “Ability to better diagnose glycemic behavior and make early interventions.”	6
Improved self-care	“I increased my care with diabetes and stopped eating foods that would change my blood glucose!” “Self-knowledge of the organism to insulin treatment.”	2
Improved quality of life	“Greater quality and freedom of life, in general” “...The use of this device has transformed my life, as after years of being diabetic and relying solely on finger-prick testing, I can now observe the extent to which my quality of life has improved with this device...” “I can only commend the initiative to provide information and technology, thereby improving and prolonging our quality of life with greater comfort.” “The sensor is an indispensable tool for reducing the disparity in quality of life that exists between a ‘normal’ individual and a person with T1DM.” “...after years of being diabetic and relying solely on finger-prick testing, I can now observe the extent to which my quality of life has improved with this device...”	5

Disadvantages:		
Themes	Reports	Reported by (n)
No disadvantages	“I did not identify a relevant disadvantage.” “I did not identify negative aspects and/or side effects.”	3
Exposure (sensor visibility)	“Giving explanations to curious people.” “The only bad thing is that people don’t know about it and think it’s a weight loss chip or a beauty chip.” “Discomfort and care with the sensor attached to the forearm.”	3
Access barriers	“Price inaccessible to most of those in need.” “Inaccessibility to support the use of the technology (without computers, without compatible smartphones).” “(...) I didn’t have any of those tools that allowed me to use it, it took me months to get a used and low-quality computer to make it work.”	4
Divergence of interstitial glucose results from capillary blood glucose	“Measurement divergent from the result measured by finger prick.” “Some sensors show values that are very different from the capillary test.”	2
Technology and data transfer	“The bad thing is having to transfer the data to the computer, having to download a program to be able to transfer the data (...).” “Occasional app failure.” “...To request that Abbot release the updated version of the Freestyle Libre as soon as possible.”	3
Sensor-related issues	“Difficulty practicing the sport I like (jiu-jitsu) due to intense physical contact, which can cause loss of the sensor by detachment. I had to adapt with special accessories.” “Precaution in contact with the equipment to avoid accidentally removing it.” “Sometimes, the sensor takes a while to detect hypo or hyperglycemia.” “I’m always afraid of damaging the sensor or it coming off... I’m apprehensive with children. A curious baby once grabbed the sensor to pull it off.” “Some sensors I’ve been using ended before the 14 days.” “When the sensor displays an error message even when installed in the location indicated by the laboratory.” “Fear of damaging it.” “The fact that the sensor’s glue is not resistant to sweat, making it difficult for those who practice physical activities.”	7

Source: author’s elaboration.

**Figure 1.**
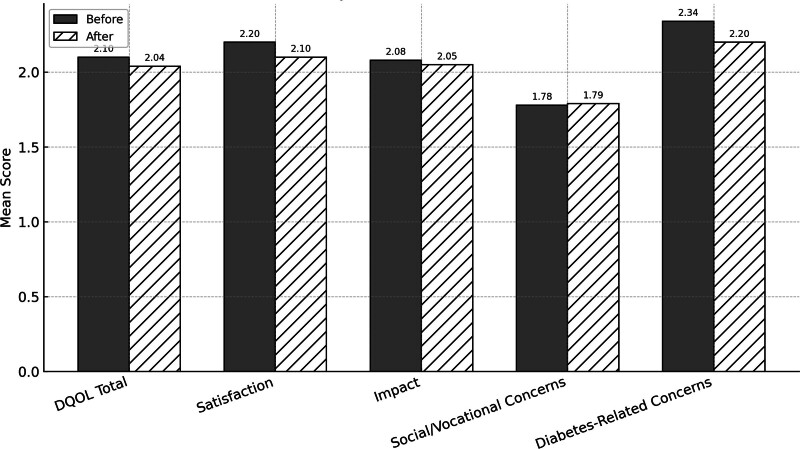
Mean scores by domain before and after technology use.

Although quantitative analysis of the DQOL-Brazil instrument demonstrated a general improvement in QoL after 3 months of FGM use, particularly in the satisfaction and diabetes-related concerns domains (Fig. 1), these changes were not statistically significant (Table 4).

**Table 4:** Mean scores before and after technology use.

	Mean score – before	Mean score – after	*P*-value
DQOL total	2.10 ± (0.46)	2.04 ± (0.47)	.273*
Satisfaction	2.20 ± (0.54)	2.10 ± (0.52)	.175*
Impact	2.08 ± (0.53)	2.05 ± (0.51)	.546*
Social/vocational concerns	1.78 ± (0.70)	1.79 ± (0.68)	.560†
Diabetes-related concerns	2.34 ± (0.70)	2.20 ± (0.72)	.075†

Source: author’s elaboration.

DQOL = Diabetes Quality of Life Measure.

* Paired *t* test.

† Wilcoxon test.

Regression analysis revealed no significant associations between QoL (total and domain scores) and sociodemographic, clinical, or prior FGM use variables both before and after FGM implementation (*P* > .05).

## 4. Discussion

Most patients in our cohort had proper glycemic control, with a mean HbA1c level of 6.9%, although 58% had HbA1c levels of ≥ 7%. This contrasts with the Brazilian Study Group on T1DM’s multicenter study, which reported suboptimal control (HbA1c > 7%) in approximately 80% of patients at public centers.^[15]^ The favorable control of our sample may be due to the SES/DF FGM selection criteria (baseline HbA1c level ≤ 8%).

The majority of patients in our sample had a diabetes duration of > 10 years. The program’s eligibility criteria mandated a minimum 2-year diabetes diagnosis, thus excluding patients with a shorter duration.

The primary insulin therapy regimen was multiple daily injections, with only 5.8% of the patients receiving continuous subcutaneous insulin infusion. Among the long-acting insulin analogs, glargine was used by 82.6% of patients, likely because of its free availability through the Unified Health System for T1DM. Daily insulin doses ranged from 0.3 to 1.48 IU/kg, with a mean of 0.74 IU/kg, consistent with typical daily insulin requirements in T1DM, which varied from 0.4 to 1.0 IU/kg.^[16]^

A mean bolus insulin percentage of 54.75% was observed, consistent with physiological insulin replacement. This pattern typically involves administering approximately 50% of the daily insulin dose as basal insulin and 50% as prandial *boluses* to manage meal-related glucose fluctuations.^[16]^

This study examined diabetes-related chronic complications and comorbidities mainly due to suboptimal glycemic control, disease duration, and genetics.^[17]^ Despite having a history of diabetes for more than a decade in many patients, 84.1% reported no complications. Retinopathy (14.5%) was the most common complication, followed by neuropathy (7.2%), consistent with the literature. The prevalence of retinopathy increases with the duration of diabetes (25% at 5 years to nearly 100% at 20 years).^[17]^ Among them, 63.8% had no comorbidities. For those who did, hypothyroidism was most common (17.4%), consistent with the known increased risk of autoimmune comorbidities, particularly thyroid disease, in T1DM compared with the general population.^[18]^ Twelve months before enrollment, 8.7% of the patients reported hypoglycemic emergencies.

Most of the patients in our study had previously used FGM. Of the new users, 37.7% came predominantly from the public health service (SUS), likely due to the high cost and of FGM and the lower socioeconomic status of SUS populations. The percentage of patients who had never used the technology previously was 37.7%, with the majority coming from the public health service (of the 26 patients who had never used the technology previously, 22 were from the SUS).

Three-month FGM analysis showed good mean TIR (68.96%) and glucose management indicator (6.69%), indicating maintained glycemic control among participants. Significant glucose instability (CV ≥ 36%) was observed in 60.9% of the sample, underscoring the complexity of T1DM management. This variability was likely influenced by episodes of hypoglycemia (<70 mg/dL and < 54 mg/dL), which were slightly outside the target range in our cohort. Although strict glycemic control is essential for preventing microvascular and macrovascular complications, it also increases the risk of hypoglycemia and may negatively impact well-being and QoL.^[19,20]^ Glycemic variability, beyond HbA1c, is increasingly recognized as an independent risk factor for complications such as cardiovascular disease, cognitive dysfunction, and reduced QoL.^[21]^ Thus, the FGM-derived metrics (particularly TIR and CV) offer valuable insights into glucose fluctuations, even among individuals with similar HbA1c levels. However, baseline glycemic metrics prior to FGM initiation were not available for most participants due to limitations in data integration across systems. As a result, the analysis of LibreView data was restricted to post-intervention descriptive evaluation, precluding formal comparisons with pre-FGM glycemic status.

CGM has been identified as a key intervention capable of altering the trajectory of type 1 diabetes, reducing mortality, and increasing healthy lifespan in Brazil.^[1]^ Other crucial interventions outlined are timely diagnosis, access to insulin and SMBG supplies, insulin pump therapy, and investments in prevention and curative research.^[1]^

Our study revealed an average sensor scan frequency of 14.27 scans per day, consistent with previous findings.^[22]^ Their research demonstrated a higher scan frequency among Brazilian users than that in a global cohort, with averages of 14 and 12 scans per day, respectively. Furthermore, they reported that increased scan frequency was correlated with lower estimated HbA1c levels, less hyperglycemia, and improved TIR.^[22]^

Patients reported significantly greater satisfaction with FGM (65.2% “very satisfied”) compared to SMBG (10.1% “very satisfied”). Additionally, 91.3% indicated that they “would always recommend” FGM to individuals with similar diabetes profiles, and 88.4% reported being “very satisfied” with continued FGM use. Higher treatment satisfaction with FGM over SMBG has also been reported in the literature.^[7]^

Despite a significant number of patients reporting no disadvantages in the qualitative assessment of FGM, the most frequently mentioned concerns were access barriers, inconsistencies between interstitial and capillary glucose measurements, and problems with sensor functionality including accidental removal and arm detachment.

FGM disadvantages such as adhesive issues, reduced accuracy, limited support, and lack of hypoglycemic alarms have been cited previously.^[23]^ The inherent 4- to 10-minute lag between capillary and interstitial glucose readings, prolonged by rapid glucose changes,^[24]^ should be acknowledged. Ideally, discrepancies should not exceed 15 mg/dL (<100 mg/dL) or 15% (>100 mg/dL). The mean absolute relative difference (MARD) quantifies the CGM accuracy as the average percentage difference between the sensor and fingerstick glucose readings; a lower MARD indicates a higher accuracy. Commercial glucose monitors generally have MARD values ranging from 10% to 12%.^[25]^ The FreeStyle Libre system has a MARD of 11.4%,^[25]^ and newer generations such as FreeStyle Libre 2 Plus, have MARDs under 8.2%.^[26]^

To address these potential technical questions, it is imperative that diabetes educators possess robust technological skills that enable them to provide accurate patient guidance. Given its integral role in disease management, diabetes education should be systematically incorporated across all tiers of healthcare provision.^[27]^

Adverse effects including premature sensor displacement and skin reactions have been documented.^[6]^ In a Belgian study, sensor complications such as early detachment, reduced confidence in readings, reduced initial accuracy, inaccuracies during hypoglycemia, rapid fluctuations, and skin irritation were reported.^[28]^ The sensor allergy rates in the published literature range from 3% to 5%, leading to discontinuation in 1% of users. In contrast, no allergic reactions were observed in this study.

The sensor visibility elicited mixed patient responses. While some reported it negatively, citing increased exposure and the need to provide explanations to curious observers, others viewed it positively, noting increased discretion during glucose measurements.

Prior research has demonstrated that FGM improves comfort, diabetes management, and QoL, particularly among young individuals and their families.^[29]^ Although studies on FGM in adults remain limited, research has reported similar positive outcomes regarding QoL, comfort, diabetes management, and self-care, along with negative perceptions related to sensor visibility.^[30]^ Notably, our study employed a substantially larger sample size compared to those used in qualitative investigations.

Although the mean total and domain scores (especially satisfaction and diabetes-related concerns) decreased after 3 months of FGM use, suggesting improved QoL, these changes were not statistically significant. While qualitative responses reflected perceived improvements in QoL, these subjective impressions were not corroborated by measurable changes in DQOL-Brazil scores, indicating a potential disconnect between self-reported experiences and standardized instrument outcomes.

Furthermore, regression analysis indicated the absence of significant associations between QoL (as assessed by the total and domain scores of DQOL-Brazil) and sociodemographic, clinical, and prior FGM use variables evaluated both pre- and post-FGM implementation (*P* > .05).

The quantitative analysis results may be partially attributed to the initially high QoL observed in our sample, which exceeded that of the DQOL-Brazil validation cohort. The baseline overall mean DQOL-Brazil score in our study was 2.10, with domain scores of 2.20 (satisfaction), 2.08 (impact), 1.78 (social/vocational concerns), and 2.34 (diabetes-related concerns). This contrasts with the validation cohort’s overall mean score of 2.46 (95% CI 2.35–2.56) and domain scores of 2.63 (95% CI 2.50–2.75), 2.29 (95% CI 2.18–2.39), 2.37 (95% CI 2.21–2.53), and 2.72 (95% CI 2.57–2.87), respectively.^[11]^

The favorable initial QoL assessment in our sample was likely influenced by patients’ proper metabolic control and adequate treatment. International studies assessing QoL with FGM using multiple instruments have shown inconsistent results. For instance, increased treatment satisfaction and reduced hypoglycemia were reported using the DTSQ, but no change was observed in general or diabetes-related QoL based on the SF-36, PAID-SF, and HFS-Worry scales.^[28]^ In contrast, intermittent monitoring in T1DM was associated with increased anxiety and depression on the HADS, despite positive findings on the Diabetes Distress Scale and overall FGM impact.^[31]^ Conversely, improved mental well-being (WHO-5) and treatment satisfaction (DTSQ) were documented, particularly among patients with T1DM,^[32]^ while findings from the FLAREN-NL4 study (n = 1365; 1054 with T1DM) showed improved general status and mental health at 6 and 12 months, but no improvement in physical health.^[7,33]^

This study has several limitations that should be acknowledged. The sample was selected through convenience sampling from a public health program with strict eligibility criteria, such as HbA1c ≤ 8% and prior adherence to SMBG, which may limit the generalizability of the findings to broader populations of individuals with type 1 diabetes (particularly those with suboptimal glycemic control or limited access to structured diabetes care). Additionally, the study did not perform an a priori sample size calculation. As it was exploratory in nature and conducted within the operational constraints of a public program, participant recruitment was limited to those who met strict inclusion criteria during the study period. As a result, the sample may have lacked sufficient statistical power to detect small but clinically meaningful changes in QoL outcomes. The absence of statistically significant differences in DQOL-Brazil scores may therefore reflect a Type II error rather than a true lack of effect. Another important limitation is the unavailability of baseline glycemic metrics for most participants, which prevented comparison with post-intervention values obtained via LibreView. This precluded temporal analyses that could have quantified changes in glycemic control attributable to FGM use. Consequently, the post-intervention glycemic data presented should be interpreted descriptively, without attributing causality to the intervention. The absence of a control group further limits causal inferences regarding the impact of FGM. The 3-month follow-up period, although sufficient to detect early impressions and satisfaction, may not capture longer-term effects on glycemic control and QoL. The study also experienced a loss to follow-up of approximately 17%, potentially introducing attrition bias if non-completers had different experiences with the technology. Moreover, the DQOL-Brazil instrument may have limited sensitivity in this context, given the high baseline scores observed, suggesting a possible ceiling effect that could obscure modest improvements. Self-reported measures of satisfaction may be influenced by social desirability bias, and some participants reported technical barriers related to data transfer and device compatibility, which may have affected adherence and data completeness. These limitations underscore the need for future studies with larger, more diverse populations, longer follow-up durations, formal sample size calculations, pre- and post-intervention glycemic comparisons, and controlled designs to fully assess the clinical and experiential impact of FGM in public healthcare settings.

## 5. Conclusion

The increasing availability of CGM has significantly transformed diabetes care. This study emphasizes the relevance of patient satisfaction, perceived autonomy, and experiential feedback as complementary outcomes to traditional clinical indicators such as HbA1c and TIR: particularly in real-world public healthcare contexts. Our findings indicate high user engagement and satisfaction with FGM use. However, the absence of statistically significant improvements in DQOL-Brazil scores highlights the limitations of the current measurement tools or study design to capture subtle but meaningful changes in QoL. Effective implementation of such technologies requires not only access but also multidisciplinary support and ongoing education. Further research should include controlled designs and economic evaluations to support broader adoption and equitable access to CGM technologies within public health systems.

The key to achieving positive clinical and QoL experiences beyond just providing access to technology lies in the effective involvement of a knowledgeable and current multidisciplinary team specializing in diabetes education. Strengthening multidisciplinary care across the entire SES/DF public health system beyond the CEDOH and HRT Endocrinology Units is crucial. Furthermore, continuing the SES/DF Program utilizing FGM or other nationally approved systems is a critical priority.

This patient-centered research offers valuable insights for clinical practice, public health policy, and future research. Future studies should prioritize the economic evaluation of expanded FGM coverage within the Unified Health System with direct benefits for QoL and the potential to improve disease progression, including reduced mortality and extended health years.

## Acknowledgments

The authors gratefully acknowledge the individuals with type 1 diabetes who generously contributed to this research and the University of Brasília for funding the publication of this study (grant number: 01/2025).

## Author contributions

**Conceptualization:** Betyna Saldanha Corbal, Erica Tatiane da Silva.

**Data curation:** Betyna Saldanha Corbal, Erica Tatiane da Silva, Camille Moreira Baptista da Silva.

**Formal analysis:** Betyna Saldanha Corbal, Erica Tatiane da Silva.

**Funding acquisition:** Rafaela Flavia da Silva Puzic.

**Investigation:** Betyna Saldanha Corbal, Erica Tatiane da Silva, Thomaz Schröder Lameirinhas, Camille Moreira Baptista da Silva, Giovanna Saldanha Ostwald Corbal.

**Methodology:** Betyna Saldanha Corbal, Erica Tatiane da Silva, Giovanna Saldanha Ostwald Corbal.

**Project administration:** Betyna Saldanha Corbal, Erica Tatiane da Silva.

**Resources:** Rafaela Flavia da Silva Puzic.

**Software:** Betyna Saldanha Corbal, Erica Tatiane da Silva.

**Supervision:** Erica Tatiane da Silva.

**Validation:** Betyna Saldanha Corbal, Erica Tatiane da Silva, Giovanna Saldanha Ostwald Corbal.

**Visualization:** Betyna Saldanha Corbal, Rafaela Flavia da Silva Puzic, Erica Tatiane da Silva, Thomaz Schröder Lameirinhas, Camille Moreira Baptista da Silva, Giovanna Saldanha Ostwald Corbal.

**Writing – original draft:** Betyna Saldanha Corbal, Rafaela Flavia da Silva Puzic, Erica Tatiane da Silva, Thomaz Schröder Lameirinhas, Camille Moreira Baptista da Silva, Giovanna Saldanha Ostwald Corbal.

**Writing – review & editing:** Rafaela Flavia da Silva Puzic, Erica Tatiane da Silva, Thomaz Schröder Lameirinhas, Camille Moreira Baptista da Silva, Giovanna Saldanha Ostwald Corbal.
